# Supplementary material for: Natural Rubber Composites Using Hydrothermally Carbonized Hardwood Waste Biomass as a Partial Reinforcing Filler- Part I: Structure, Morphology, and Rheological Effects during Vulcanization
Source: Polymers (Basel). 2023 Feb 26;15(5):1176. doi: 10.3390/polym15051176 (PMC10007617; doi:10.3390/polym15051176)
Supplement: Supplementary file 1 [file polymers-15-01176-s001.zip › polymers-2235362-supplementary.pdf]

SUPPLEMENTARY INFORMATION

Natural Rubber Composites Using Hydrothermally Carbonized Hardwood Waste Biomass as a Partial Reinforcing Filler- Part I: Structure, Morphology, and Rheological Effects during Vulcanization

Jelena Lubura [1,*], Libor Kobera [2], Sabina Abbrent [2], Ewa Pavlova [2], Beata Strachota [2], Oskar Bera [1], Jelena Pavličević [1], Bojana Ikonić [1], Predrag Kojić [1] and Adam Strachota [2,*]

[1] Faculty of Technology Novi Sad, University of Novi Sad, Bulevar cara Lazara 1, 21000 Novi Sad, Serbia
[2] Institute of Macromolecular Chemistry, Czech Academy of Sciences, Heyrovskeho nam. 2, CZ-162 00 Praha, Czech Republic
* Correspondence: jelenalubura@uns.ac.rs (J.L.); strachota@imc.cas.cz (A.S.)

1 Estimation of specific surface from particle size

A simple estimation of the specific surfaces of materials as function of particle size can be done using the model of a cube being divided into smaller cubes (**Scheme**).

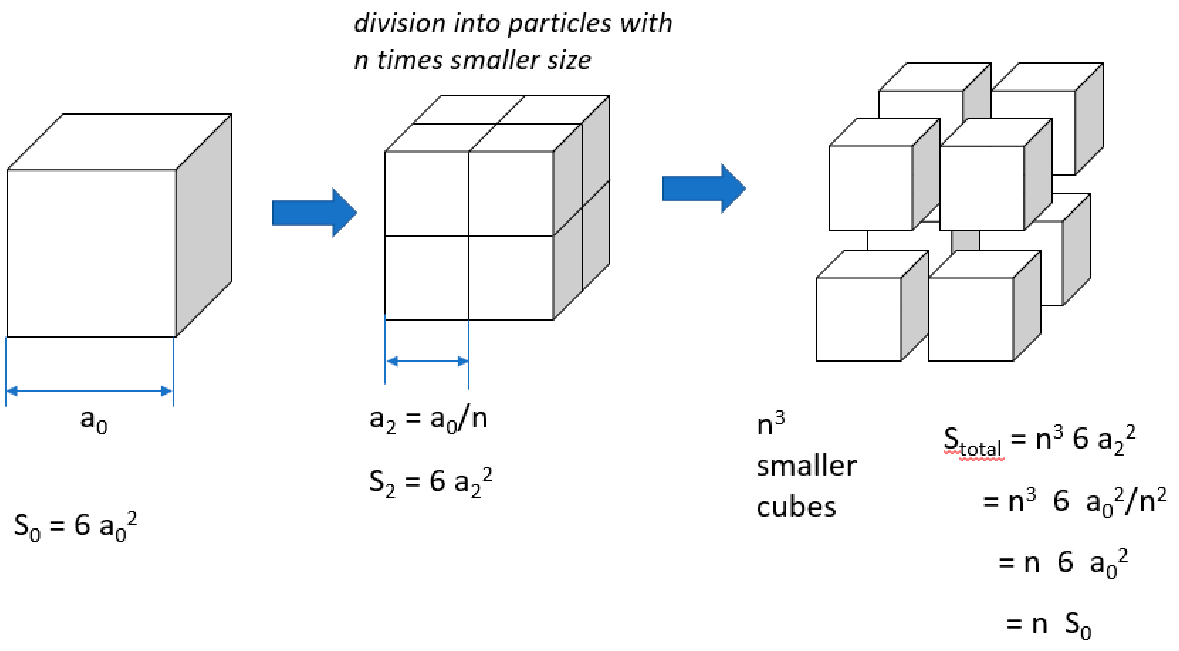

Scheme S1: Division of a cube with edge length a_0 into smaller ones with n times shorter edge length.

It can be seen from Scheme S1 that if a cube of the edge length a_0 is divided into smaller ones, whose edge length is n times shorter, then the total surface area of the resulting small cubes is n times larger than the surface area S_0 of the precursor cube. (The total volume $V_0 = a_0^3$ does not change).

Hence, if the density and filler amount are constant, n times smaller particles mean n times higher surface area of the smaller filler.

If both the sizes and densities (ρ) of the particles are different, then the size (a_{0i}) of the original (precursor) piece (monolithic cube) of a given filler amount (e.g. m_{0i} = 1 g) is different for every filler. It holds:

$V_{0i} = a_{0i}^3$

$S_{0i} = 6\ a_{0i}^2$

$\rho_i = m_{0i} / a_{0i}^3$

hence

$a_{0i} = (m_{0i} / \rho_i)^{1/3}$

$S_{0i} = 6\ (m_{0i} / \rho_i)^{2/3}$
and
$a_{02} / a_{01} = (m_{02}/ \rho_2)^{1/3} / (m_{01} / \rho_1)^{1/3} = (\rho_1 / \rho_2)^{1/3}$
$S_{02} / S_{01} = 6\ (m_{02}/ \rho_2)^{2/3} / 6\ (m_{01} / \rho_1)^{2/3} = (\rho_1 / \rho_2)^{2/3}$

A less dense filler (e.g. filler "2" with ρ_2, a_{02}, and S_{02}) has to be added in a larger volume than the denser filler "1", if the same wt.% (or phr) have to be achieved.

If both filler 1 and filler 2 are added in the same amount (in g) and are divided into the same number pf particles, than the particles of filler 2 are larger, and their total surface area also is larger than in case of filler 1, as given by the above equations for the ratios a_{02} / a_{01} and S_{02} / S_{01}.

For the fillers CB and HC, it can be calculated
with $m_{0CB} = m_{0HC} = 1$ g and
with
$\rho_{CB} = 2969$ kg/m^3 (as determined experimentally in this work) and
$\rho_{HC} = 1407$ kg/m^3 (as determined experimentally in this work):

for CB:
a_{0CB}(1g cube) = 0.00696 m (= 6.96 mm)
S_{0i}(1g cube) = 0.0002904 m^2 (= 2.904 cm^2)
particle size (a_{2CB}) found by TEM: 30–60 nm
size factor (a_{0CB}/a_{2CB}): 1.16*10^5 – 2.32*10^5
theoretical specific surface area: 33.7 – 67.4 m^2/g
difference from experimental (77.8 m^2): factor 1.15–2.31 (average: 1.73)

for HC:
a_{0CB}(1g cube) = 0.00892 m (= 8.92 mm)
S_{0i}(1g cube) = 0.000478 m^2 (= 4.78 cm^2)
particle size (a_{2HC}) found by TEM: 0.5–3 μm
size factor (a_{0HC}/a_{2HC}): 2 975 – 17 800
theoretical specific surface area: 1.42 – 8.53 m^2/g
difference from experimental (21.4 m^2): factor 2.51–15.1 (average: 8.79)

HC vs. CB:
the density factor: $\boldsymbol{S_{0HC} / S_{0CB}}$ = 1.64
size ratio (from TEM): $\boldsymbol{a_{0HC} / a_{0CB}}$ = ca. 17–50
theoretical specific surface area ratio if densities of CB and HC were the same: 17–50
theoretical specific surface area ratio, corrected by density factor: 27–82

experimentally found (BET) ratio of specific surfaces:
77.8 m^{2}g^{-1} / 21.4 m^{2}g^{-1} = 3.63
difference from theory: factor 7.5–22.6

2 Fillers: Specific surface analysis

2.1 sample "CB"

Summary Report

Surface Area
Single point surface area at P/Po = 0.300303893: 76.2684 m^2/g
BET Surface Area: 77.8403 m^2/g

Options Report

Analysis Conditions

Preparation
Evacuation rate: 100.0 mmHg/min
Evacuation time: 15.00 min

Free Space
Free-space type: Measured

Po and Temperature
Po and T type: Most recent measured Po value. Entered temperature value.
Temperature: 77.150 K

Analysis Method
Analysis Mode: Equilibration
Equilibration time: 15 s

Degas Conditions

Sample Prep: Stage	Temperature (°)	Ramp Rate (°/min)	Time (min)
1	90	10	0

Adsorptive Properties
Adsorptive: Nitrogen
Maximum manifold pressure: 1050.00 mmHg
Non-ideality factor: 0.0000620
Density conversion factor: 0.0015468
Molecular cross-sectional area: 0.162 nm^2

Experiment
Started: 11.12.2022 9:43:06odp.
Completed:
Report Time: 11.12.2022 13:58:53odp.
Free Space Diff.: -0.2489 cm^3 Sample
Free Space Type: Measured
Evac. Rate: 100.0 mmHg/min
Gemini VII 2390 V1.01 (V1.01 a)

Analysis Adsorptive: N2
Equilibration Time: 15 s
Sat. Pressure: 735.844 mmHg
Mass: 0.0518 g
Sample Density: 2.969 g/cm^3
Micromeritics Instrument Corporation

Isotherm Tabular Data: sample "CB"

Saturation Pressure (mmHg)
735.843811

Relative Pressure (P/P_0) ( )	Absolute Pressure (mmHg)	Quantity Adsorbed (cm^3/g STP)	Elapsed Time (h:min)
0.049898117	36.71722	16.6576	1:02
0.07472054	54.98265	17.5963	1:07
0.079783256	58.70802	17.7969	1:11
0.099543311	73.24833	18.4357	1:16
0.124927246	91.92694	19.3207	1:21
0.139744301	102.83	19.7229	1:26
0.142832311	105.1023	19.8242	1:31
0.149716719	110.1681	20.0235	1:35
0.174945411	128.7325	20.884	1:40
0.199839583	147.0507	21.7011	1:45
0.225042997	165.5965	22.525	1:50
0.235945476	173.619	22.834	1:55
0.249926054	183.9065	23.3253	2:00
0.275288475	202.5693	24.1804	2:05
0.300303893	220.9768	25.0395	2:09
0.329161111	242.2112	26.0502	2:14
0.350476254	257.8958	26.8253	2:19
0.422393335	310.8155	29.3764	2:24
0.450421095	331.4396	30.3889	2:29
0.515674517	379.4559	32.8786	2:34
0.550580227	405.1411	34.2731	2:39
0.608902988	448.0575	36.8241	2:45
0.701359689	516.0912	41.8803	2:51
0.740250322	544.7086	44.5928	2:57
0.794441802	584.5851	49.6104	3:04
0.840416919	618.4156	55.8577	3:11
0.886894356	652.6157	66.7701	3:22
0.904951076	665.9026	73.7827	3:33
0.939989623	691.6855	101.5176	3:58

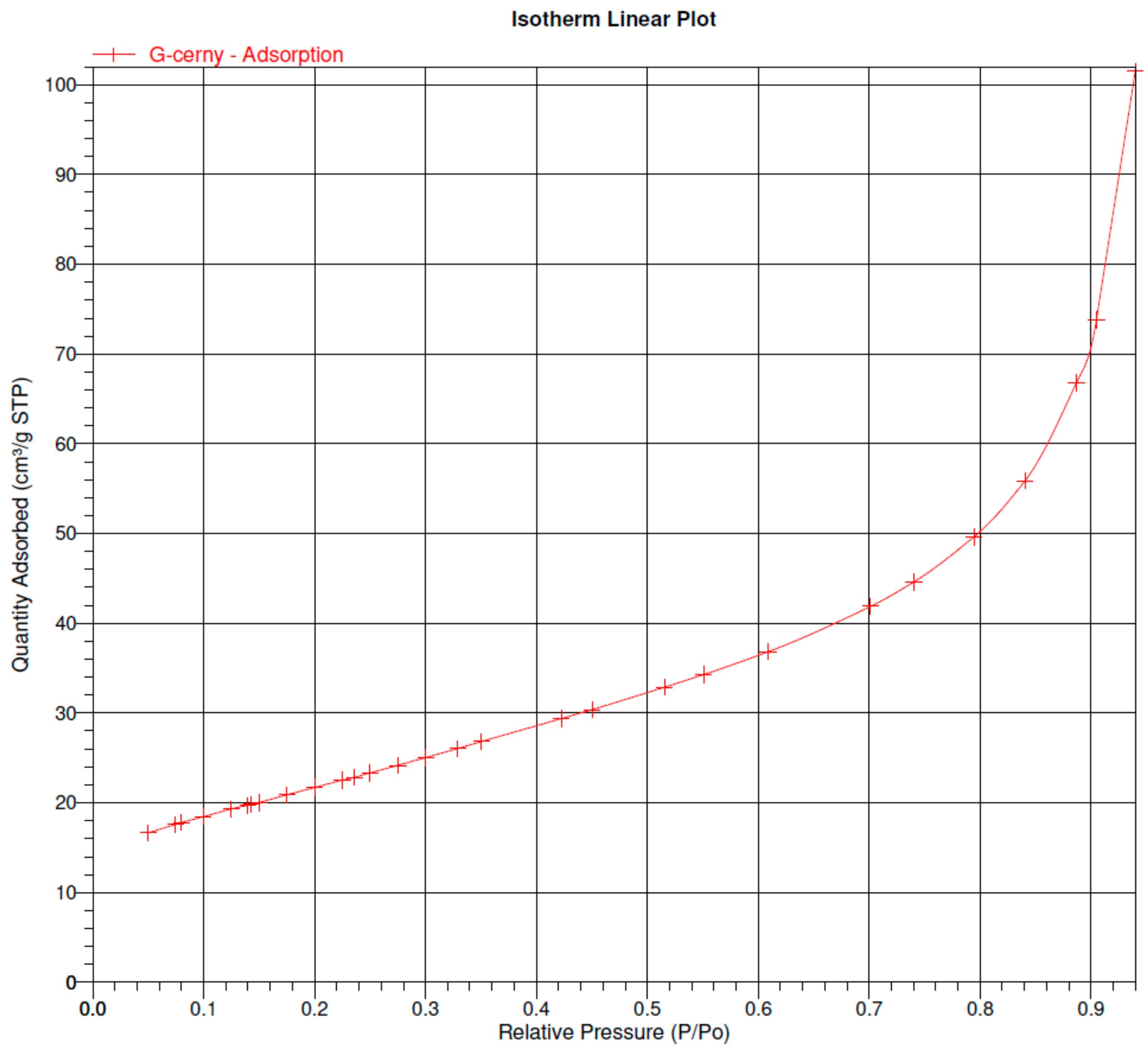

Figure S1. Adsorption isotherm (of nitrogen) on the sample "CB".

BET Surface Area Data: sample "CB"

BET Surface Area: 77.8403 ± 0.1566 m^2/g

Slope: 0.055478 ± 0.000111 g/cm^3 STP

Y-Intercept: 0.000447 ± 0.000020 g/cm^3 STP

C: 125.055284

Qm: 17.8812 cm^3/g STP

Correlation Coefficient: 0.9999741

Molecular Cross-Sectional Area: 0.1620 nm^2

Relative Pressure (P/P_0)	Quantity Adsorbed (cm^3/g STP)	1 / [Q(P/P_0 - 1)] ( )
0.049898117	16.6576	0.003153
0.07472054	17.5963	0.004589
0.079783256	17.7969	0.004872
0.099543311	18.4357	0.005996
0.124927246	19.3207	0.007389
0.139744301	19.7229	0.008236
0.142832311	19.8242	0.008406
0.149716719	20.0235	0.008794
0.174945411	20.884	0.010153
0.199839583	21.7011	0.011509
0.225042997	22.525	0.012892
0.235945476	22.834	0.013524
0.249926054	23.3253	0.014285
0.275288475	24.1804	0.015709
0.300303893	25.0395	0.017141

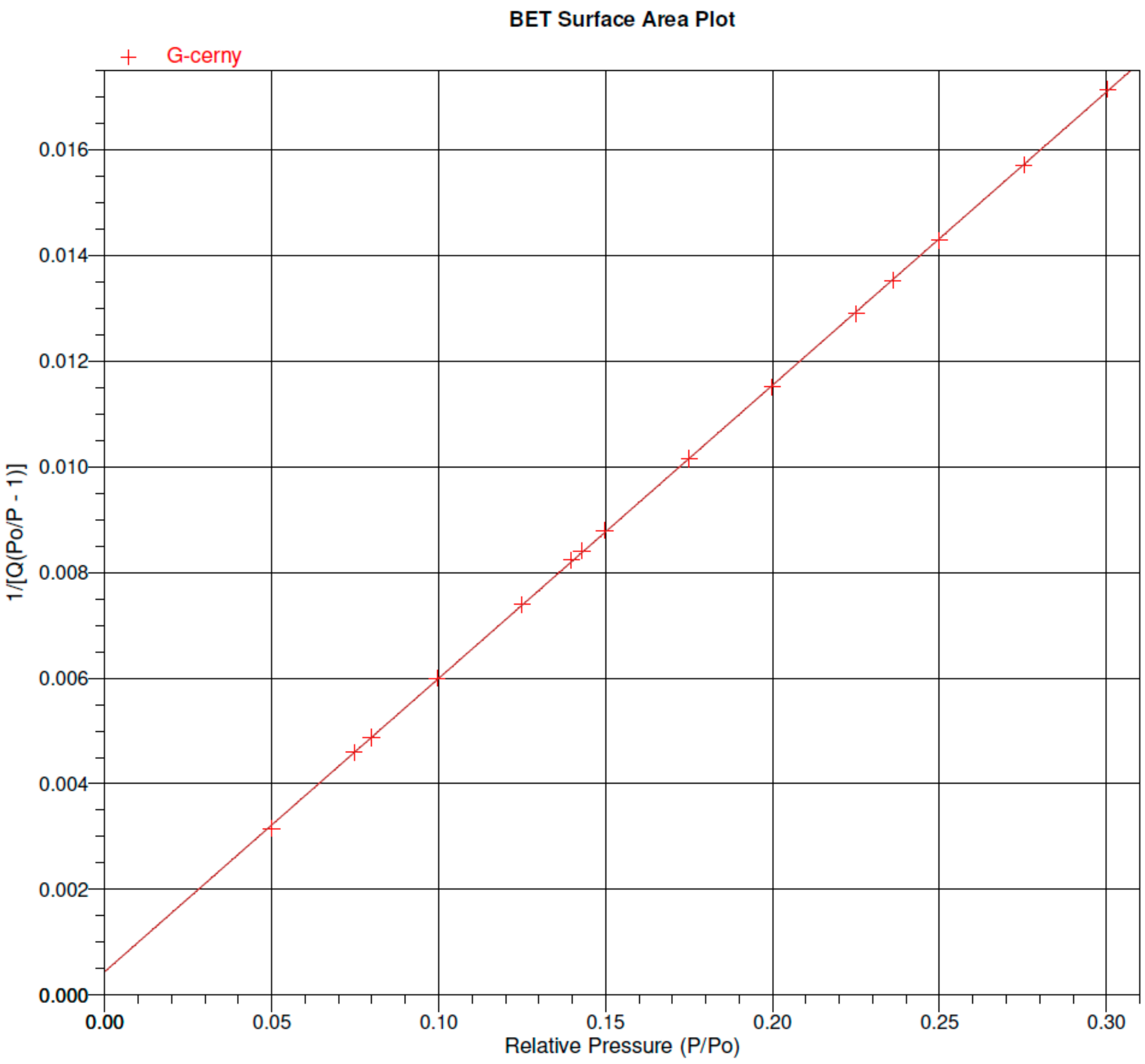

Figure S2. BET-plot of the adsorption isotherm (of nitrogen) on the sample “CB”.

2.2 sample "HC"

Summary Report
Surface Area
Single point surface area at P/P_0 = 0.300333577: 16.1976 m^2/g
BET Surface Area: **21.3628 m^2/g**

Options Report

Analysis Conditions

Preparation
Evacuation rate: 100.0 mmHg/min
Evacuation time: 15.00 min

Free Space
Free-space type: Measured

P_0 and Temperature
P_0 and T type: Most recent measured P_0 value. Entered temperature value.
Temperature: 77.150 K

Analysis Method
Analysis Mode: Equilibration
Equilibration time: 15 s

Degas Conditions

Sample Prep: Stage	Temperature (°)	Ramp Rate (°/min)	Time (min)
1	90	10	0

Adsorptive Properties
Adsorptive: Nitrogen
Maximum manifold pressure: 1050.00 mmHg
Non-ideality factor: 0.0000620
Density conversion factor: 0.0015468
Molecular cross-sectional area: 0.162 nm^2

Experiment
Started: 12.12.2022 7:12:35pm
Completed: 12.12.2022 10:53:53pm
Report Time: 12.12.2022 10:53:54pm
Free Space Diff.: -0.1764 cm^3
Free Space Type: Measured
Evac. Rate: 100.0 mmHg/min
Gemini VII 2390 V1.01 (V1.01 a)

Analysis Adsorptive: N_2
Equilibration Time: 15 s
Sat. Pressure: 737.604 mmHg
Sample Mass: 0.0067 g
Sample Density: 1.407 g/cm^3
Micromeritics Instrument Corporation

Isotherm Tabular Data: sample "HC"

Saturation Pressure (mmHg)
737.603760

Relative Pressure (P/P_0) ( )	Absolute Pressure (mmHg)	Quantity Adsorbed (cm^3/g STP)	Elapsed Time (h:min)
0.049548202	36.54694	1.1705	1:02
0.074690135	55.09172	1.9263	1:06
0.079673647	58.76758	2.0759	1:11
0.099589798	73.45781	2.7953	1:16
0.124712823	91.98865	3.2422	1:21
0.139911925	103.1996	3.7847	1:26
0.142721506	105.2719	3.2455	1:31
0.149936357	110.5936	3.5417	1:37
0.174458312	128.6811	3.6504	1:42
0.199597349	147.2238	3.8079	1:46
0.224892283	165.8814	4.6242	1:51
0.235823424	173.9442	4.451	1:55
0.24984764	184.2886	4.657	2:00
0.275206808	202.9936	4.7967	2:04
0.300333577	221.5272	5.318	2:09
0.329279038	242.8775	5.8377	2:13
0.350309394	258.3895	6.1167	2:18
0.422373954	311.5446	7.0002	2:23
0.450678326	332.422	7.9227	2:28
0.515632773	380.3327	8.7645	2:32
0.550645309	406.1581	9.2317	2:37
0.608948688	449.1628	9.9155	2:41
0.701460252	517.3997	10.9698	2:46
0.740700709	546.3436	11.329	2:50
0.794822547	586.2641	12.0648	2:55
0.84085884	620.2206	12.5536	2:59
0.887693403	654.766	13.6197	3:03
0.905954787	668.2357	13.8525	3:08
0.94085702	693.9797	14.0482	3:13
0.96489429	711.7097	14.4939	3:17
0.977974001	721.3573	14.4711	3:22
0.981893355	724.2482	14.8229	3:28
0.985918046	727.2169	14.8436	3:34
0.987254425	728.2026	14.0991	3:40

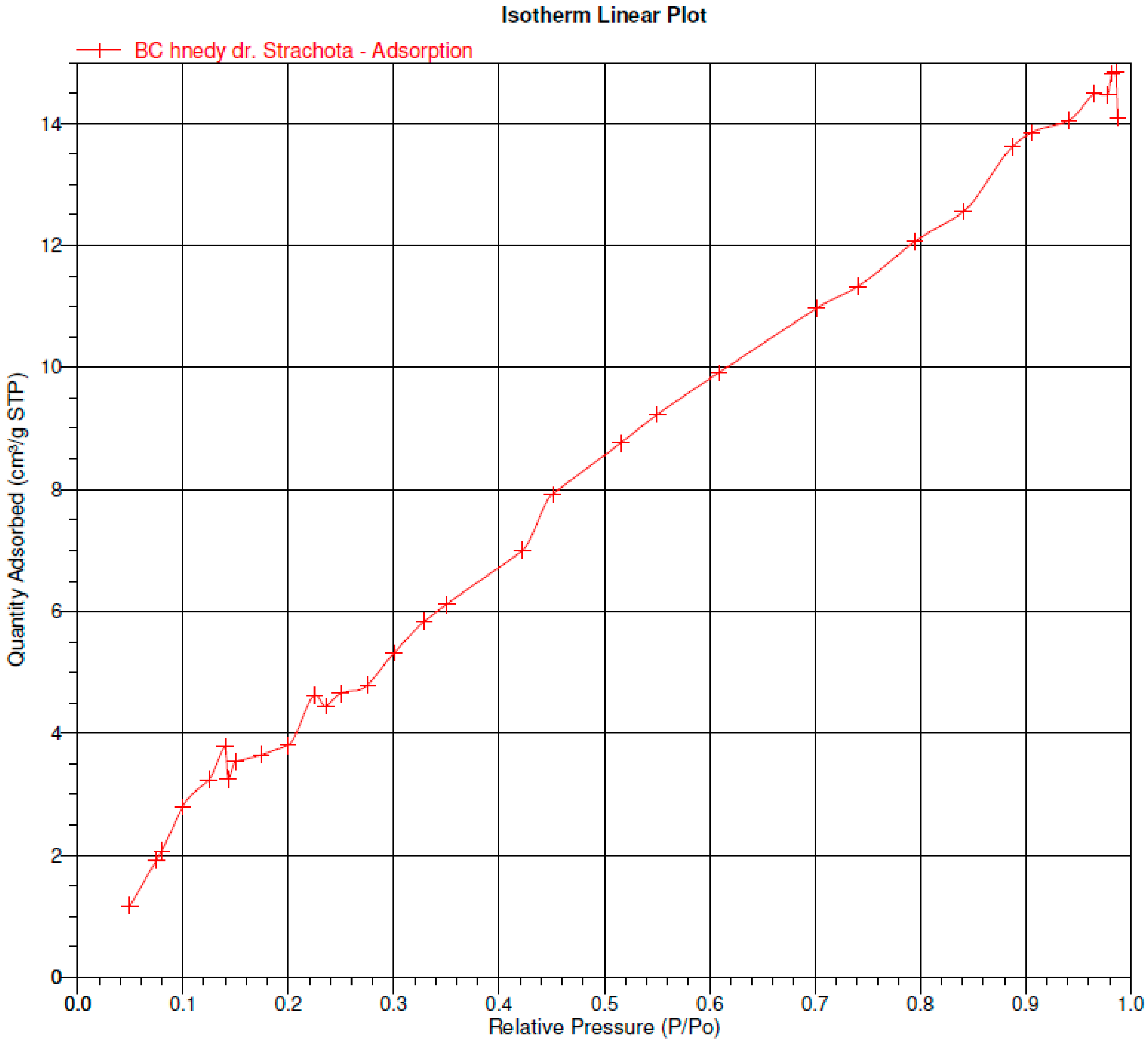

Figure S3. Adsorption isotherm (of nitrogen) on the sample "HC".

BET Surface Area Data: sample "HC"

BET Surface Area: 21.3628 ± 1.6445 m^2/g

Slope: 0.177420 ± 0.015428 g/cm^3 STP

Y-Intercept: 0.026355 ± 0.002837 g/cm^3 STP

C: 7.731971

Qm: 4.9074 cm^3/g STP

Correlation Coefficient: 0.9542024

Molecular Cross-Sectional Area: 0.1620 nm^2

Relative Pressure (P/P_0)	Quantity Adsorbed (cm^3/g STP)	$1 / [Q(P/P_0 - 1)]$ ( )
0.049548202	1.1705	0.044538
0.074690135	1.9263	0.041903
0.079673647	2.0759	0.041703
0.099589798	2.7953	0.039569
0.124712823	3.2422	0.043946
0.139911925	3.7847	0.042982
0.142721506	3.2455	0.051296
0.149936357	3.5417	0.049802
0.174458312	3.6504	0.057891
0.199597349	3.8079	0.065488
0.224892283	4.6242	0.062744
0.235823424	4.451	0.069332
0.24984764	4.657	0.071519
0.275206808	4.7967	0.079159
0.300333577	5.318	0.080716

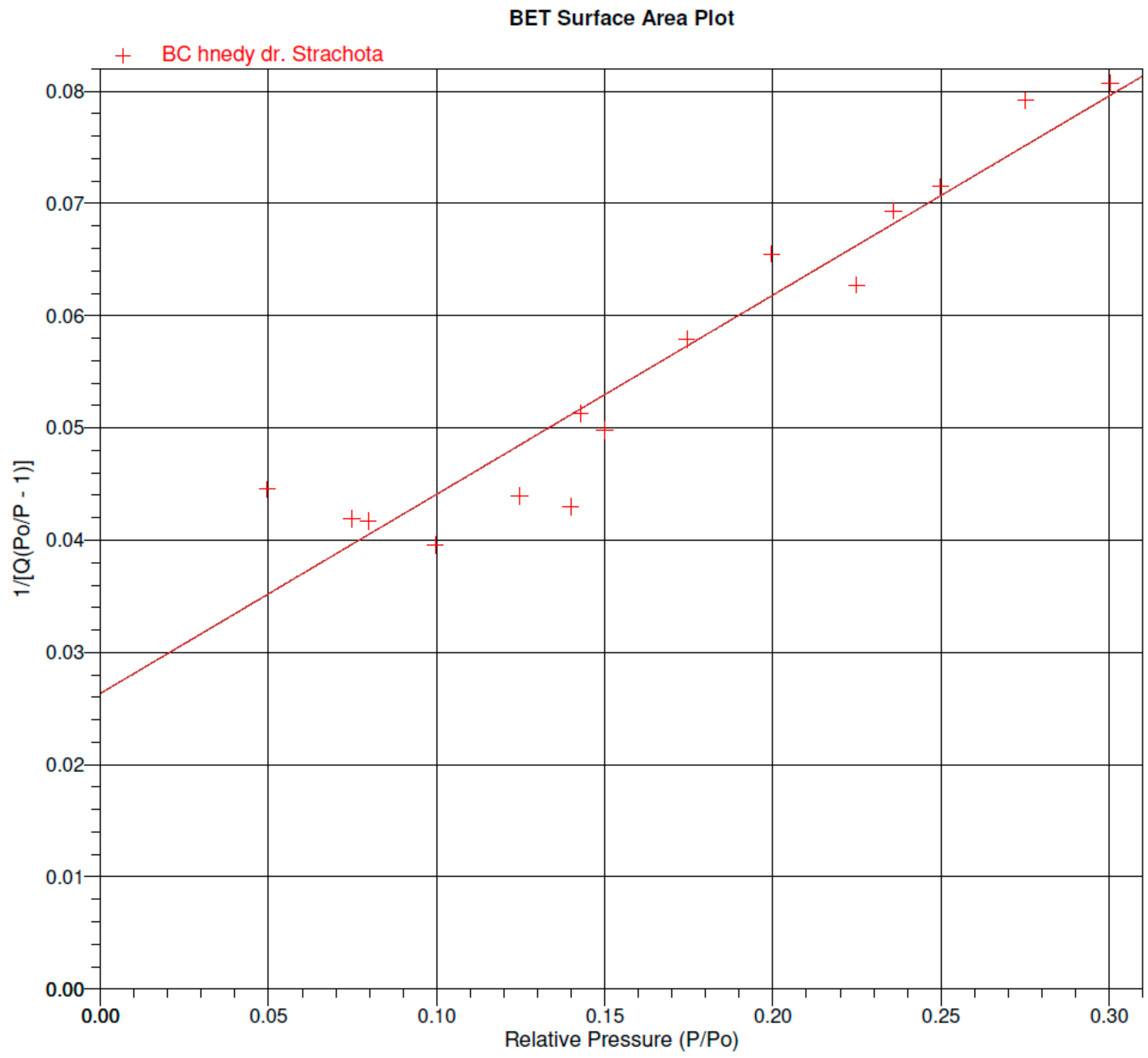

Figure S4. BET-plot of the adsorption isotherm (of nitrogen) on the sample "HC".

3 Nanocomposite Morphology: TEM/SEM

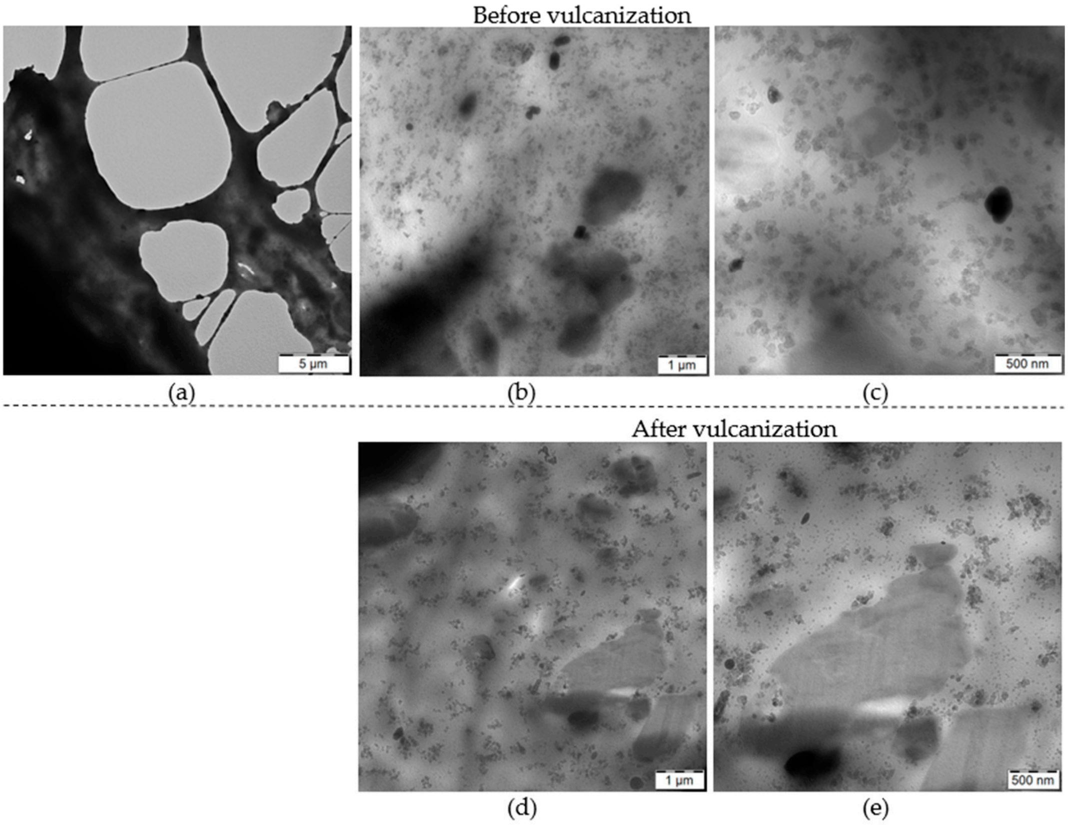

Figure S5. TEM images of the sample filled with 40 phr (25.16 wt.%) of hydrochar and 10 phr (6.29 wt.%) of carbon black: (a, b, c) unvulcanized state: sample CB10; (d, e) vulcanized state: sample VCB10; (a): 'global view' – 5 μm scalebar; (b, d): 1 μm scalebar; and (c, e): 500 nm scalebar.

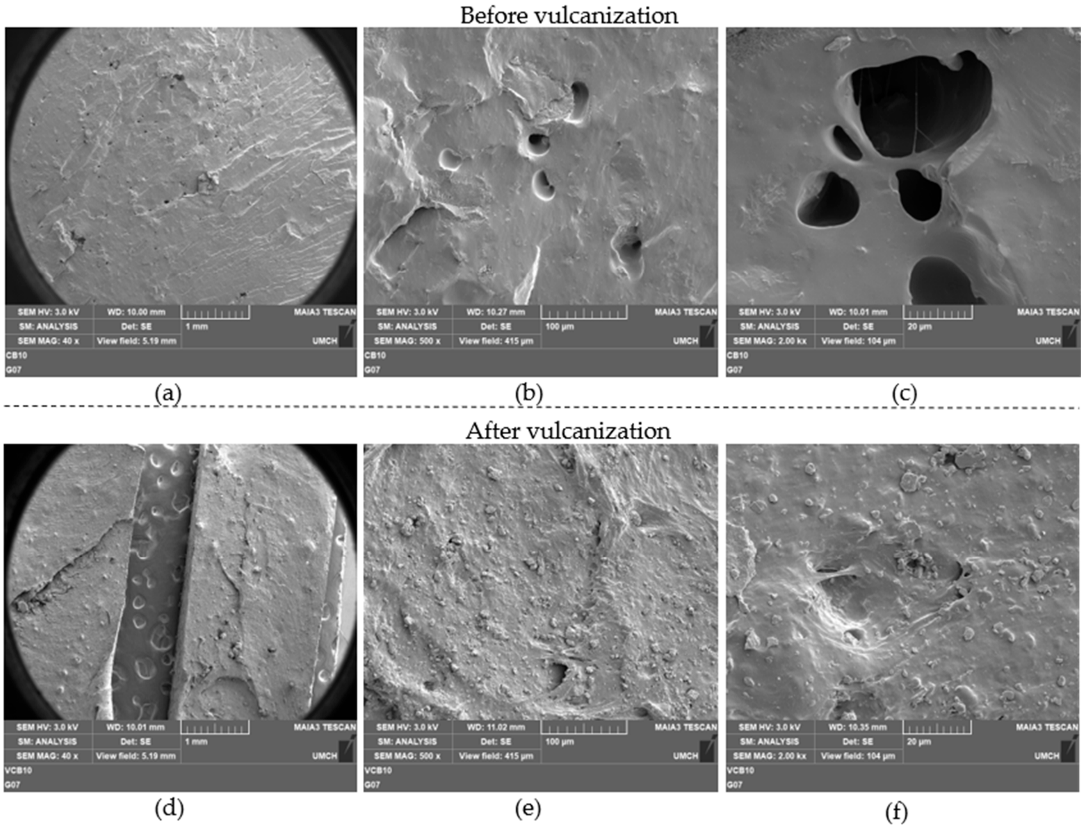

Figure S6. SEM images of the sample filled with 40 phr (25.16 wt.%) of hydrochar and 10 phr (6.29 wt.%) of carbon black: (a, b, c) unvulcanized state: sample CB10; (d, e, f) vulcanized state: sample VCB10; (a, d): really global view – 1 mm scalebar; (b, e): 100 μm scalebar; and (c, f): 20 μm scalebar.

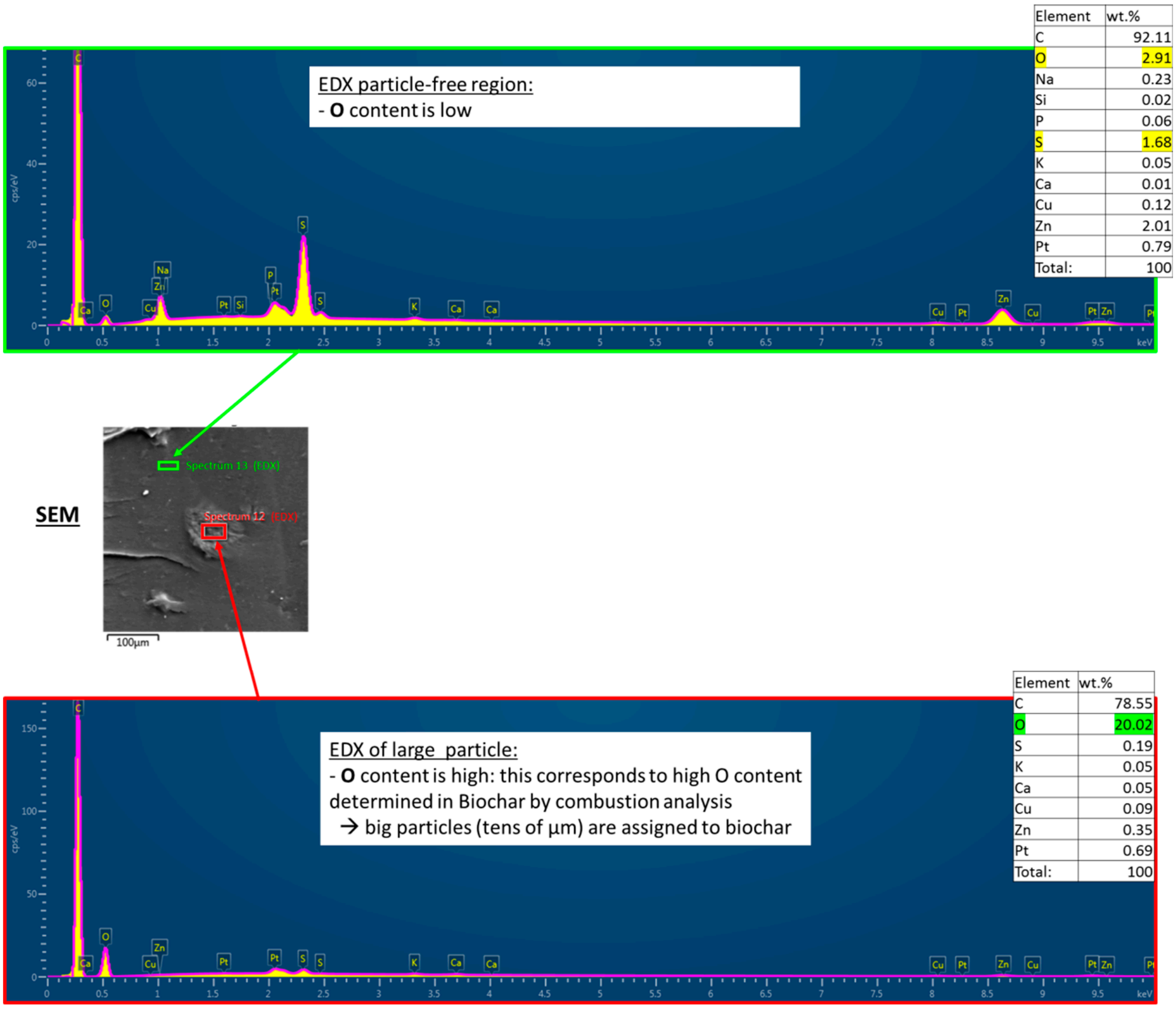

Figure S7. EDX elemental analysis from the SEM image of the sample VCB40: top: hydrochar-free domain; bottom: hydrochar grain; in the hydrochar-free matrix (top) sulphur can clearly be detected, following trace elements also are visible: Zr (from ZrO2 balls in ball mill), Pt (from applied metal coating used in SEM for better sharpness), Cu (from Cu grid on which the SEM sample was placed), Na, Ca, K (minerals from wood which stayed in the hydrochar).

4 FTIR of nanocomposites

Unvulcanized and vulcanized samples were analysed using FTIR spectroscopy and the results are presented in the Figure S8. The unvulcanized and vulcanized samples with the same ratio of the fillers (HC/CB) are showing nearly identical FTIR spectra (see Figure S8a vs. b). On the other hand, the influence of the presence of the graphitic CB filler (which does not display any intense peaks, as shown in the Main Manuscript) on the intensity of the IR peaks of the composites clearly can be seen: All the observable IR peaks which originate mainly from the natural rubber strongly decrease in intensity with increasing CB content, which could be assigned to indiscriminate reflection on the graphitic CB phase. At high contents of hydrochar, its characteristic groups of peaks in the region 1700 down to 1000 cm^{-1} appear to prominently contribute to the FTIR pattern of the composites (see e.g. the overlay in Figure S8).

The following assignments of the registered peaks can be made (on the example of sample VCB10, see Figure S8b and c): The characteristic vibrational absorption bands of C–H stretching of natural rubber (and also of hydrochar) are found at 2962, 2917 and 2852 cm^{-1}, assigned to aliphatic groups (a weak signal of unsaturated C-H stretching of rubber and hydrochar also can be seen at 3037 cm^{-1}). The small absorption peak at 1656 cm^{-1} corresponds to C=C stretching in rubber and is overlaid with C=C peaks of HC. The absorption bands, detected at 1442 cm^{-1} and 1373 cm^{-1} are related to the C-H bending of the groups $-CH_2-$ (rubber and HC) and $-CH_3-$ (predominantly from rubber), respectively. In view of the prominent broad peak around 3300 cm^{-1}, the presence of water in KBr can be noted, while the occasionally observed peak near 2350 cm^{-1} is caused by carbon-dioxide from the atmosphere.

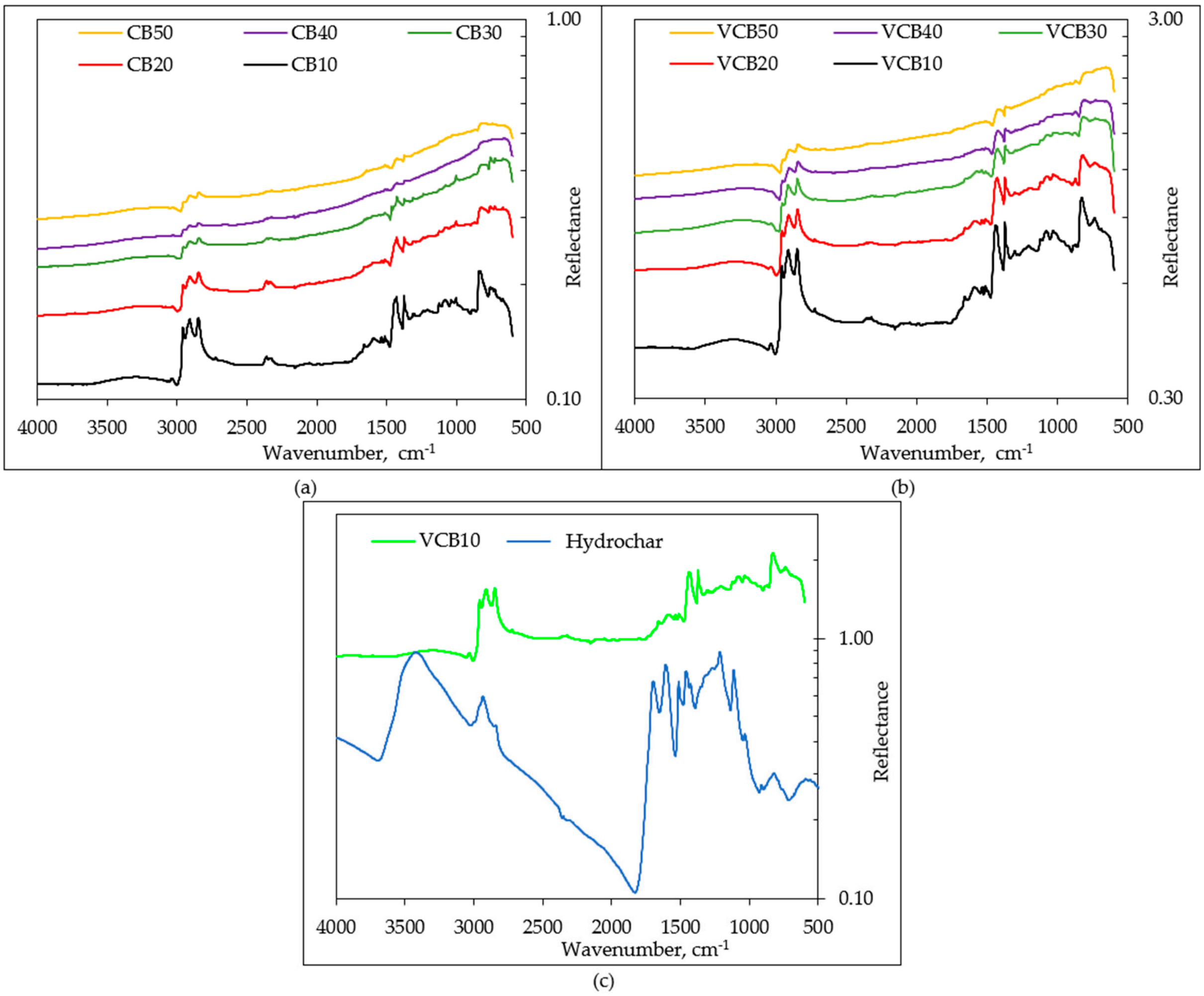

Figure S8. FT-IR spectra of a) unvulcanized, b) vulcanized natural rubber samples, and c) overlay of hydrochar and VCB10 spectra.
